# Metabolic and hormonal responses to a single session of kumite (free non-contact fight) and kata (highly ritualized fight) in karate athletes

**DOI:** 10.1007/s11332-012-0132-7

**Published:** 2012-11-17

**Authors:** S. Benedini, S. Longo, A. Caumo, L. Luzi, P. L. Invernizzi

**Affiliations:** 1 Department of Sport, Nutrition and Health Sciences, Università degli Studi di Milano, Milan, Italy; 2 Research Center of Metabolism IRCCS Policlinico San Donato Milanese, Milan, Italy; 3 Research Center of Metabolism, Piazza Edmondo Malan, 20097 San Donato Milanese, Milan, Italy

**Keywords:** Glucose, Catecholamines, Insulin, Testosterone

## Abstract

**Background:**

Several studies report martial arts as a good model for investigating neuroendocrine responses to competitive fighting. However, little is known on the metabolic responses elicited by elite athletes during fighting. In particular, the metabolic picture in elite athletes of martial arts is little known.

**Aim:**

In the present study, our aim was to investigate the acute effects of a session of karate practice on the glucose-insulin system.

**Subjects and methods:**

Ten healthy individuals (6M/4F; BMI: 22.1 ± 0.7 kg/m^2^; 21.9 ± 1.1 years, mean ± SE) who practice karate in national or international competitions were enrolled. All participants completed two experimental trials in a randomised-crossover fashion. A basal blood sample was collected from each athlete to assess plasma glucose, insulin, cortisol, testosterone and catecholamines, before karate training session. In two separate days, another blood sample was collected from each participants after 3 min of real fighting (*kumite*) and 3 min of ritualized simulation of combat (*kata*).

**Results:**

In both trials, plasma glucose resulted to be higher at the end the of performance compared to the basal (*p* < 0.001 after *kumite* and *p* < 0.02 after *kata*). In contrast, insulin was similar in the basal and after physical activity in the two trials. Catecholamines were higher after kata and kumite sessions with respect to the basal values (*p* < 0.04) and, in particular, epinephrine post-kumite values were much greater than those measured after kata.

**Conclusions:**

Our results indicate that unlike performances of karate (*kumite* and *kata*) elicit different plasma glucose increases. In particular, we found that glucose and epinephrine concentrations increased more after kumite than after kata.

## Introduction

There is much evidence showing important alterations of hormone profile in athletes of martial arts [1, 2]. In particular, a number of studies carried out on elite athletes have tried to confirm the hormonal pathway found in other animal models with likewise trends of responses in serum testosterone and cortisol levels [3]. Other studies investigated exercise-induced hormonal changes to find new insights about physiology of martial arts [4, 5]. Furthermore, the practice of martial arts might be helping to lower hypertension and reduce the risk of cardiovascular disease mortality by improving the cardiorespiratory effort adaptation, increasing muscular capillarization, oxidative enzyme activity and maximum oxygen consumption [6].

Glucose metabolism was poorly investigated in the aforementioned studies. In fact, significant hormonal alterations can affect metabolic profiles importantly. For example, cortisol is a counter-regulatory hormone that is capable of rising plasma glucose, whenever abundantly released by the adrenal cortex [7]. From an endocrinological point of view, the response to competitive situations is elicited even before the competitive activity starts (anticipatory hormonal response) and it has been emphasized in previous works [8].

Indeed, a study conducted in diabetes patients showed benefits on glycemic control and insulin resistance after practising Tai Chi on a regular base [9]. In fact, physical activity increases body sensitivity to leptin and insulin, promoting the sense of satiety via interleukin-6 pathway. These findings support the hypothesis that appetite-suppressive actions of exercise may be mediated by hypothalamus [10], which controls on its turn the pituitary output by secreting specific chemical signals toward pituitary’s front lobe [11]. Hence, physical activity is capable of crosstalking with the autonomic nervous system. In fact in vivo epinephrine in liver promotes hepatic glucose output by activating glycogenolysis and accelerating glyconeogenesis, while simultaneously inhibiting glycogen synthesis [12, 13].

To investigate the glycemic and hormonal responses elicited by karate, ten young elite of karate athletes performed two types of exercises used in karate competitions in two different days.

## Materials and methods

### Selection criteria

All subjects (six males and four females) were healthy and lean (BMI: 22.1 ± 0.7 kg/m^2^), and on a stable diet, with normal glucose tolerance (according to ADA). All athletes (the age and anthropometric parameters data are summarized in Table 1) practice karate in national or international competitions and with a mean training load of 10–12 h per week. The subjects were recruited from Milan in Italy. All subjects signed a written informed consent prior to participation, according to the Declaration of Helsinki. All the procedures used complied with the Good Clinical Practice (GCP) principles.

**Table 1:** Anthropometrical and clinical characteristics of the study subjects

Variable	Mean ± SE
Sex (M/F)	6 M/4 F
Age (years)	21.9 ± 1.1
BMI (kg/m^2^)	22.2 ± 0.7
Fasting glucose (mmol/l)	4.9 ± 0.2
Insulin (pmol/l)	25.8 ± 2.3

### Study protocol

The study was carried out at Dojo-Anshin Kai-Milan. Each participant of the study visited the Dojo in two separate days (at 9.00 a.m.) for the execution of the two exercises (*kata* and *kumite*) in a randomized-crossover fashion. The Kata (KA) session is a simulation of combat consisting in a sequence of highly ritualized and stereotyped pattern of fighting, comprehending puches and kicks. The Kumite (KU) session is a non-contact fighting with punches and kicks against a real opponent. Each performance lasted 3 min and was supervised by a federal judge according to the international rules of the World Karate Federation. On each day, two blood samples were drawn from each participant to assess both the pre- and post-test concentrations of plasma glucose, insulin, cortisol, testosterone and catecholamines. The pre-test sample was collected 30 min before physical exercise, while post-test sample was collected within 10 min after the physical exercise. In the present study, our aim was to investigate the acute effects of a session of karate practice on the glucose–insulin system.

### Analytical methods

Hormonal responses were recorded immediately before and after the exercise bouts by means of blood sample analysis. In addition to plasma glucose, plasma insulin, cortisol, testosterone, epinephrine and norepinephrine were also measured.

The total amount of blood drawn from each participant was about 22.5 ml. All blood samples were placed on ice until plasma or serum were separated by centrifugation at 4 °C (within 1.5 h from sampling). All plasma and serum aliquots were frozen at −60 °C for later analysis. All the samples were measured in duplicate. Plasma glucose was measured with a glucose analyzer (Beckman Instruments, Fullerton, CA). Aliquots of blood for measurement of catecholamines were collected in test tubes containing EDTA; all the samples were kept on ice and the plasma prepared by centrifugation at 4 °C (within 1.5 h from sampling). Epinephrine and norepinephrine levels were measured by enzyme immunoassay method (ELISA; DRG Instruments, Marburg, Germany). Free insulin was dosed by a highly specific two-site monoclonal antibody-based immunosorbent assay (ELISA; Dako Diagnostics, Cambridgeshire, UK). Plasma cortisol and total testosterone were measured with commercial ELISA kit.

### Statistical analysis

Data were expressed as mean ± SEM. Normality of data distributions was checked by graphical methods and by the Shapiro–Wilk’s test. Epinephrine and norephinephrine concentrations were subjected to a logarithmic transformation to induce normality in their distribution and allow the use of parametric statistical tests. Each participant was characterized by four data sets, two for kata (pre- and post-test) and two for kumite (pre- and post-test). Student’s paired *t* test was used to compare KA pre-test versus post-test measurements, as well as KU pre-test versus post-test measurements. For each metabolic (or endocrine) variable displaying a significant change both after *kata* and *kumite*, another Student’s paired *t* test was used to determine whether the size of the change induced by KA differed from the change induced by KU. Data analysis was carried out with SPSS^®^ 18.0 software. A *p* value lower than 0.05 was considered statistically significant.

## Results

**Fig. 1:**
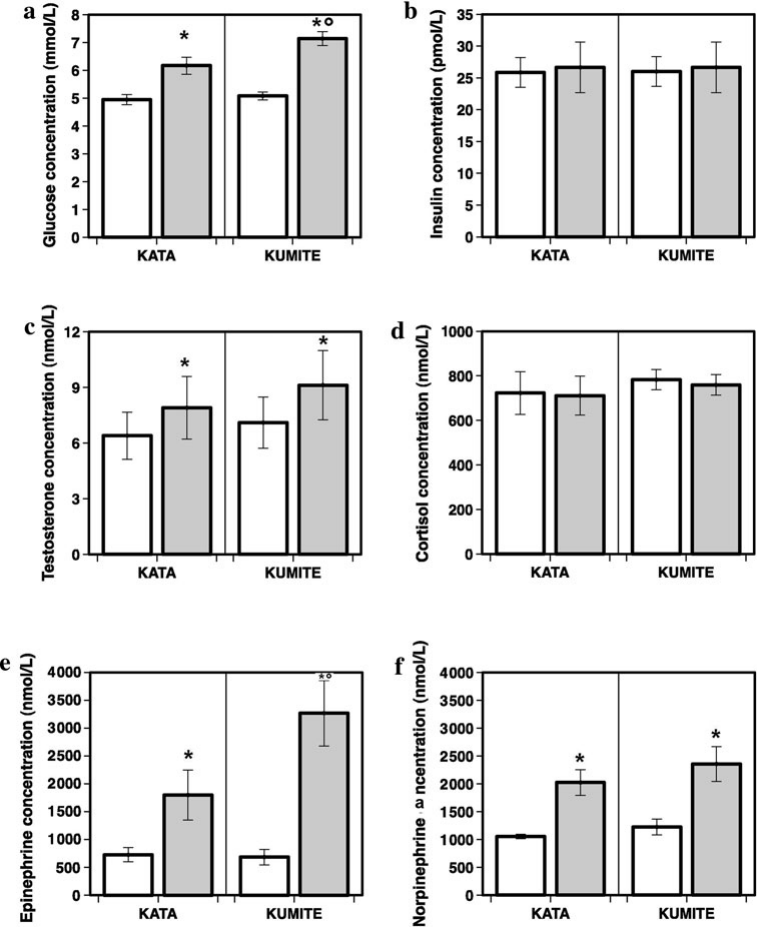
**a** Plasma glucose in the basal condition (*white bar*) and after kata or kumite (*gray bars*). **b** Plasma insulin in the basal condition (*white bar*) and after kata or kumite (*gray bars*). **c** Total testosterone in the basal condition (*white bar*) and after kata or kumite (*gray bars*). **d** Plasma cortisol in the basal condition (*white bar*) and after kata or kumite (*gray bars*). **e** Plasma epinephrine in the basal condition (*white bar*) and after kata or kumite (*gray bars*). **f** Plasma norepinephrine in the basal condition (*white bar*) and after kata or kumite (*gray bars*). *p < 0.02 compared to basal °*p* < 0.04 kumite vs kata

The plasma concentrations (mean ± SEM) of glucose, insulin, cortisol, testosterone, epinephrine and norepinephrine measured before and after the KA and KU exercises are displayed in Fig. 1.

### Plasma glucose

Fasting plasma glucose was similar and within the normal range in all subjects (4.9 ± 0.2 mmol/l) at baseline. Values of plasma glucose significantly increased to 7.1 ± 0.3 mmol/l after KU (*p* < 0.002), and to 6.2 ± 0.3 mmol/l after KA (*p* < 0.0001) compared to basal values. Moreover, the post-*kumite* plasma glucose concentration was significantly higher than that of post-*kata* (*p* < 0.02).

### Plasma insulin

The basal plasma insulin was similar in all subjects and within the low range of normality (25.8 ± 2.3 pmol/l). Surprisingly, after both *Kumite* and *Kata* the values of plasma insulin were very similar to the basal condition and no significant differences were found between exercises (26.6 ± 4 and 26.6 ± 4 pmol/l, *p* = n.s., *kumite* and *kata*, respectively).

### Steroid hormones

Plasma cortisol was within the normal range in the basal condition (752.5 ± 52 mmol/l). No significant differences were found between basal and post-exercise in both *Kumite* and *Kata* (759.1 ± 46 and 711.1 ± 87 mmol/l, respectively), nor between types of exercise (*p* = 0.50). Testosterone was similar in the basal condition (6.7 ± 1.4 mmol/l). However, Testosterone increased after both *kumite* (9.1 ± 1.9 mmol/l, *p* < 0.01) and *kata* (7.9 ± 1.7 mmol/l, *p* = 0.01) compared to the basal condition. No significant differences were found between diverse kind of performance.

### Catecholamines

The basal values of epinephrine were in the upper range of normality (702 ± 127 pmol/l), probably as a response to the imminent combat. Epinephrine increased from basal levels after both *kumite* (3,269 ± 590 pmol/l, *p* < 0.001) and *kata* (1,871 ± 367 pmol/l, *p* < 0.007) and with significant differences between the two karate routines (*p* < 0.04). Basal values of norepineprine were within the normal range (1,149 ± 75 pmol/l). Likewise, norepinephrine increased after both *kumite* (2,356 ± 313 pmol/l, *p* < 0.002) and *kata* (2,023 ± 229 pmol/l, *p* < 0.002) but without significant differences between the two karate routines performance (*p* = 0.29).

## Discussion

In this study, we compared the hormonal milieu before and after 3 min of two types of karate practice, namely *kata* and *kumite*. We found that both KA and KU produced significant increases in glucose, testosterone and cathecolamines levels, without appreciable changes in insulin and cortisol concentrations. In addition, we found that glucose and epinephrine concentrations increased more after kumite than kata. After KU, glucose was high with normal levels of plasma insulin, probably provoked by hormone profile changes. Different hormones interfering with glucose profile were studied, such as cortisol, testosterone and catecholamines. The increase in plasma glucose after KU was higher than the one measured after KA. However, cortisol, testosterone and norepinephrine levels remained similar after physical performances. Only epinephrine was significantly higher after *kumite* respect to the values recorded after *kata*. In fact, epinephrine is the most important counter regulatory hormone that is capable to increase glycogenolysis, gluconeogenesis, lipolysis and amino acids uptake only after 3 min of heavy combat [7]. Previous studies have proposed that catecholamines, specifically epinephrine, are the main mediators of the increment in hepatic glucose output during intense exercise [14, 15]. Furthermore, testosterone was higher after both exercises. In contrast, plasma cortisol concentrations were similar to the basal condition. This difference between steroid hormones could be explained by an acute activation of gonads before activation of the hypothalamic–pituitary–adrenal axis. Interestingly, norepinephrine did not increase as much as epinephrine after *kumite.* However, this catecholamine is primarily secreted by the sympathetic nervous system as neurotransmitter, therefore it is a hormone secreted also by the adrenal medulla in response to splanchnic stimulation, like it occurs during hypotension. In fact, since the athlete is like in a condition of real danger during a heavy combat, the secretion of epinephrine seems to be induced by a *fight*-*or*-*flight* reaction. Given that aggressions are quite infrequent in the Western countries in the 21st Century, the martial art fight seems an interesting model of *fight*-*or*-*flight* condition.

During kumite, high levels of epinephrine allowed an increased plasma glucose availability which could be responsible for a temporary insulin resistance condition. As an explanation for such a phenomenon, high efficiency of muscle contraction is met by an increase of plasma glucose availability so that human genotype would maintain unaltered energy balance. These findings confirm the involvement of epinephrine in the regulation of glucose homeostasis, first as a physiological stressor signal, and second, participating as a counter-regulatory hormone released to fulfill energy balance [7–13, 16].

So far, previous investigations [1, 5] have been focusing on the hormones testosterone and cortisol with the purpose of examining the endocrine effects of martial arts. To the best of our knowledge, no data are available in the literature on the effects of martial arts on the glucose–insulin system and a marked hyperglycemic response was not undiscovered yet. Despite the small sample size of the study, due to the high peculiarity of élite athlete matched for technical ability, BMI, age, gender and willingness to give blood samples, a strong correlation between plasma glucose increase and epinephrine was found.

The elevation in glucose concentration observed after both the *kumite* and *kata* practice may result from an increase in endogenous glucose production and a decrease in peripheral glucose clearance. Both such effects may be mediated by epinephrine. In contrast, testosterone increases after both KA and KU. In fact, exercise-induced increment of testosterone is well known in literature [17, 18]. Given the metabolic alterations seen after practising kumite, this kind of karate should be discouraged in dysmetabolic individuals or in subjects prone to develop metabolic alterations. Recognized that the better metabolic profile after practice of kata, this kind of karate should be encouraged in dysmetabolic individuals or in subjects prone to develop metabolic alterations respect kumite. On the contrary, no contraindications exist for performing ritualized simulation of combat (kata) given the slight increase of glucose registered in the study (after kata combat). Particularly, exercise training induces changes in metabolic energy homeostasis in skeletal muscles and improves insulin action by reducing the accumulation of incompletely oxidized fatty acids [19]. Indeed, the present findings should not be applied to amateur athletes who obtain benefits from any kinds of physical exercise, with improvement of blood pressure cardio-respiratory fitness, as well as insulin sensitivity increase.

## Abbreviations

BMI: Body mass index
ADA: American Diabetes Association
